# Marchiafava-Bignami Disease in a Patient With Polysubstance Use Disorder

**DOI:** 10.7759/cureus.59730

**Published:** 2024-05-06

**Authors:** Natalia Chalupczak, Connor Cole, Nita Lohala

**Affiliations:** 1 Chicago Medical School, Rosalind Franklin University of Medicine and Sciences, North Chicago, USA; 2 Internal Medicine, Ascension Saint Joseph Hospital, Chicago, USA

**Keywords:** neuroimaging, mri, demyelination, polysubstance abuse, marchiafava-bignami disease

## Abstract

Marchiafava-Bignami disease (MBD) is a rare demyelinating disease associated with chronic alcohol use and/or malnutrition leading to vitamin deficiency. Clinical presentation is diverse and can range from mild neurological deficits of dysarthria and confusion to severe symptoms such as coma or even death. Diagnosis is made using imaging modalities including magnetic resonance imaging (MRI) and computed tomography (CT) with the rise in technological advances placing MRI as the most sensitive and specific imaging technology for diagnosis. Classic MBD imaging demonstrates demyelination and necrotic damage of the corpus callosum. While MBD is a well-documented neurologic complication of chronic alcoholism, its occurrence and presentation in the context of concurrent polysubstance abuse remain underexplored. We outline the case of a 27-year-old male with polysubstance use disorder presenting with subacute neurological deterioration and demyelination of the splenium of the corpus callosum.

## Introduction

Marchiafava-Bignami disease (MBD) is a rare neurological disorder primarily linked with chronic alcoholism. It is characterized by demyelination in the corpus callosum, though it may also affect other areas of the brain [1]. Although the link between the pathogenesis of MBD and excessive alcohol consumption has been well established, the precise mechanisms through which alcohol and potentially other factors contribute to the disease's development remain an area of ongoing research. There have been a few cases described in the literature of non-alcoholic patients presenting with MBD, suggesting that alcohol is not the only cause of this demyelination [2]. Clinically, MBD presents a spectrum of symptoms ranging from mild cognitive impairments such as memory loss and confusion to severe neurological deficits including tremors, speech difficulties, and changes in gait and consciousness. MBD's variability makes comprehensive neurological assessments and the utilization of advanced imaging techniques, such as magnetic resonance imaging (MRI), for accurate diagnosis critical. Given its rarity and the complexity of its presentation, there is a significant need for a deeper understanding of this disease's pathophysiology and for the development of more effective management strategies. 

## Case presentation

A 27-year-old male with a prior medical history of chronic polysubstance abuse disorder, epilepsy, major depressive disorder, and latent tuberculosis infection presented to our institution for altered mental status. The patient was initially admitted to the recovery center for opioid, alcohol, and benzodiazepine withdrawal but was transferred to the telemetry unit due to an acute change in mental status two days after admission. He experienced dysarthria, altered sensorium, decreased consciousness, and ataxia and was subsequently transferred. He had been chronically drinking alcohol since the age of 16. He began abusing drugs at the age of 18 with brief periods of sobriety spanning from three to six months duration at a time. He had a period of complete abstinence from all substances from the ages of 24 to 26 before resuming heavy drug use of cocaine, oxycodone, heroin, and Xanax at the age of 26. The latest consumption of these substances was reported to be the day of admission to the recovery unit, two days prior to hospital admission. Of note, the patient was also admitted eight years ago with complaints of altered mentality and new-onset seizures where an electroencephalogram (EEG) was positive for epileptic discharge and the patient was discharged on Keppra. Subsequently, he was seen by a neurologist and was told to stop the Keppra and had stopped taking it since then but also had not had any additional seizure episodes. 

Initial work-up on admission at the recovery center was pertinent for a urine toxicology screen that was positive for benzodiazepines, fentanyl, cocaine, and opiates (Table 1). The blood alcohol level was noted to be less than 10 mg/dL. While in the recovery unit, the patient was being managed with scheduled and as-needed phenobarbital, folic acid, thiamine, multivitamin tablets for alcohol withdrawal, and sublux as needed for opioid withdrawal. The following morning after admission, he was noted to be lethargic and confused and was subsequently transferred to the telemetry unit for further monitoring. On physical examination upon transfer, the patient was found to be in a state of altered sensorium (AOx2-3, GCS 15). He was drowsy in mentality and showed disoriented features in time and place, as well as some upper and lower extremity ataxia. Over the following two days, the patient's condition worsened acutely with a continued decrease in consciousness and subsequent lack of response to tactile stimuli. The patient became obtunded and unarousable. He was at times able to localize pain and opened his eyes occasionally to verbal stimulation. Spontaneous speech was seldom observed, and the speech was slurred and incomprehensible. All these signs and symptoms indicated a hemispheric disconnection.

**Table 1:** Urine toxicology screen results

Urine toxicology screen	Result	Threshold
Benzodiazepines	Positive	50-300 ng/mL
Amphetamines	Negative	500-1000 ng/mL
Cocaine	Positive	150-300 ng/mL
Cannabinoids (THC)	Negative	50 ng/mL
Opiates	Positive	2000 ng/mL
Phencyclidine (PCP)	Negative	25 ng/mL
Barbiturates	Negative	200-300 ng/mL
Buprenorphine	Negative	5-10 ng/mL
Fentanyl	Positive	1 ng/mL

The laboratory data revealed leukocytosis, hyponatremia, hyperglycemia, and mild hypokalemia (Table 2 and Table 3). The patient's liver functions were slightly abnormal but renal functions were all in normal range. Thyroid-stimulating hormone (TSH) level was notable for being slightly low as well.

**Table 2:** Serum laboratory test results Na+: sodium; K+: potassium; Ca2+: calcium; TSH: thyroid-stimulating hormone; ALT: alanine aminotransferase; AST: aspartate aminotransferase; BUN: blood urea nitrogen

Serum	Obtained values	Reference range
Na+	128 mEq/L	136-146 mEq/L
K+	3.1 mEq/L	3.5-5.0 mEq/L
Ca2+	9.8 mg/dL	8.5-10.2 mg/dL
Albumin	4.1 g/dL	3.4-5.4 g/dL
Glucose	106 mg/dL	70-100 mg/dL
Lactate	66 U/L	45-200 U/L
TSH	0.164 μU/mL	0.27-4.2 μU/mL
ALT	60 U/L	10-40 U/L
AST	68 U/L	12-38 U/L
Creatinine	0.71 mg/dL	0.6-1.2 mg/dL
Ammonia	34 u/dL	15-60 u/dL
BUN	14 mg/dL	7-18 mg/dL

**Table 3:** Hematologic laboratory test results

Hematologic	Obtained values	Reference range
Hemoglobin, blood	14 g/dL	13.5-17.5 g/dL
Hematocrit	42%	41-53%
Erythrocyte count	4.8 mcL	4.3-5.9 mcL
Leukocyte count (WBC)	15.9 mcL	4.5-11 mcL

**Figure 1:**
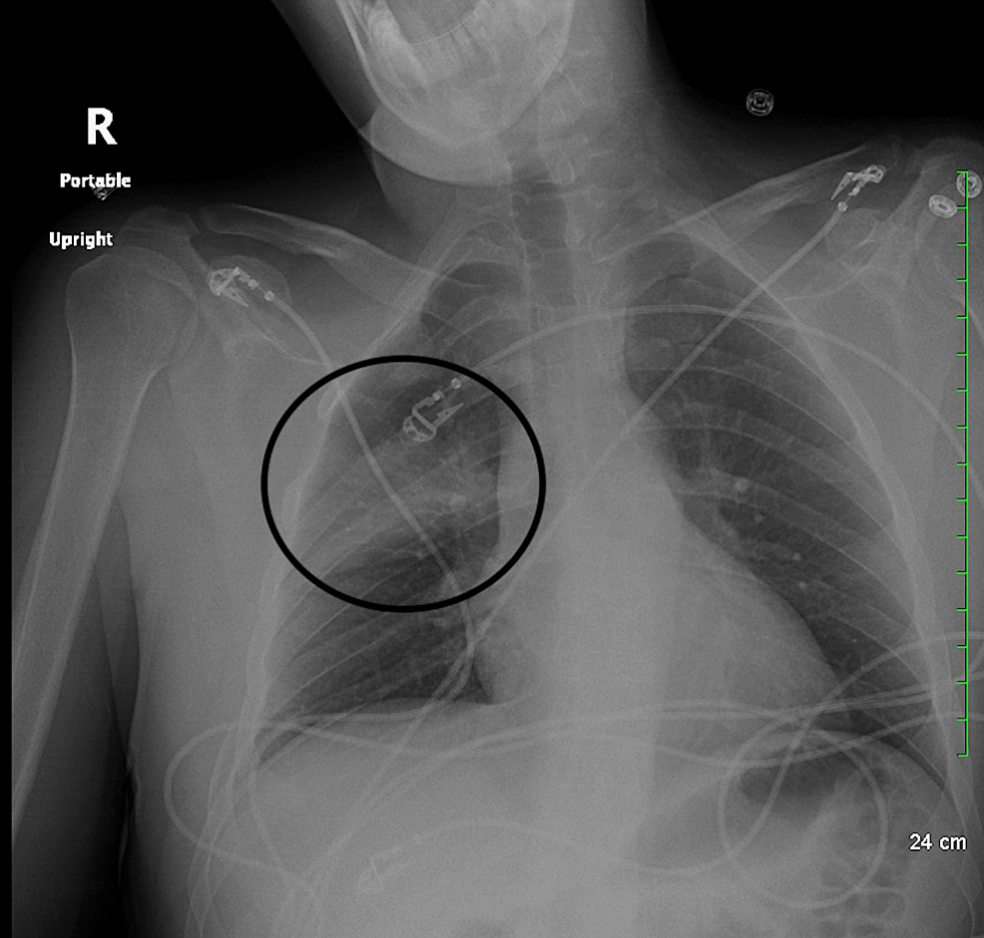
Chest X-ray showing right upper lobe infiltrates

**Figure 2:**
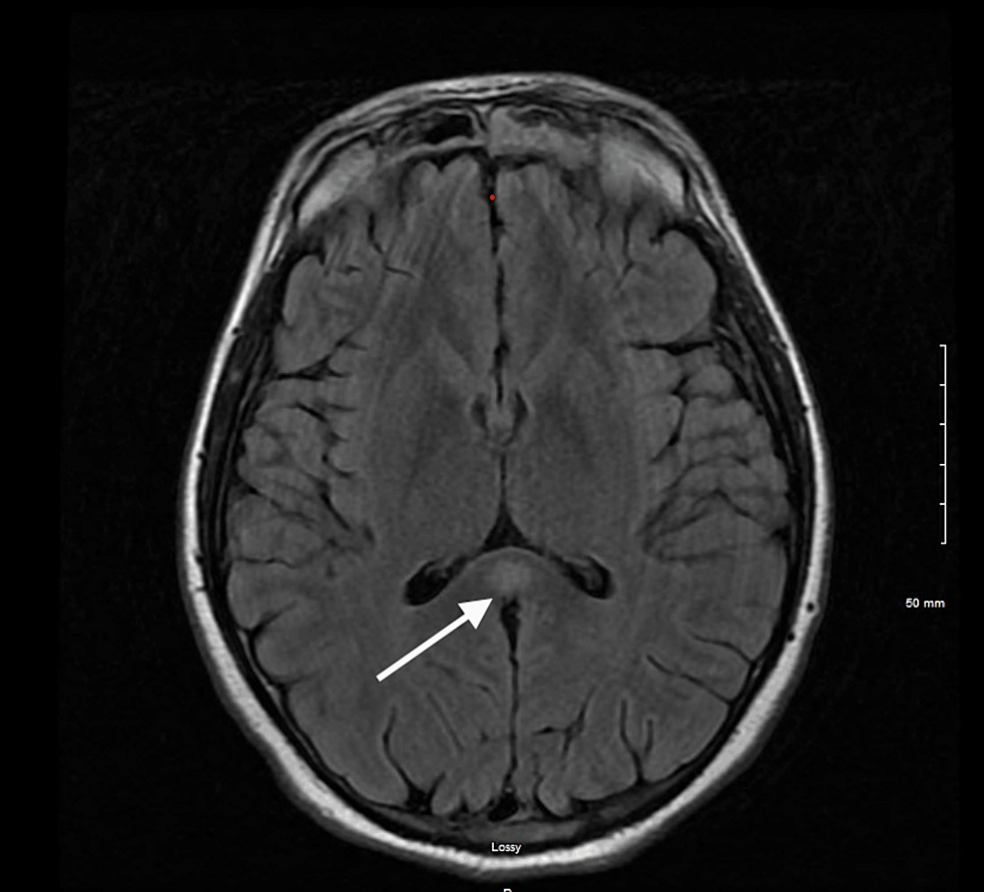
Axial T2-FLAIR MR image showing a high signal intensity area in the central part of the corpus callosum FLAIR: fluid-attenuated inversion recovery

**Figure 3:**
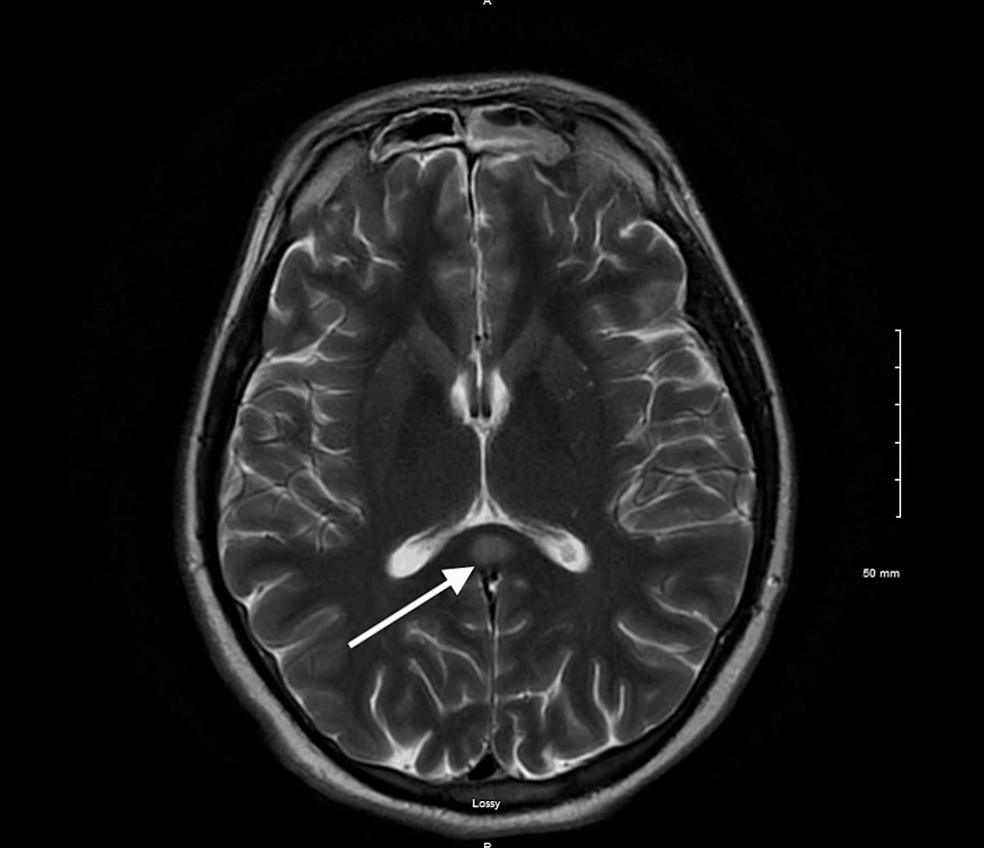
T2-weighted axial MR image showing symmetrical hyperintensity in the region of the corpus callosum, particularly concentrated in the splenium

**Figure 4:**
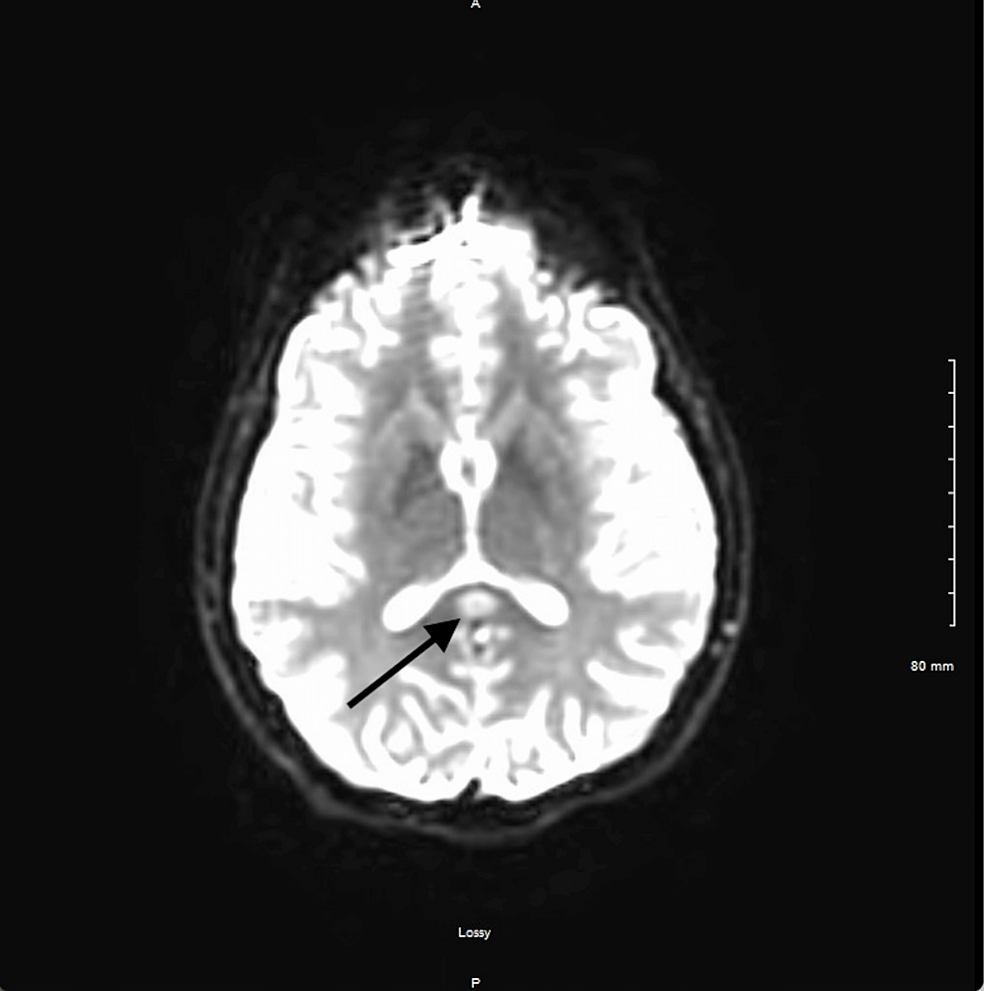
Diffusion-weighted MR image demonstrating hyperintensity in the corpus callosum

Chest X-ray showed right upper lobe infiltrates indicative of aspiration pneumonia (Figure 1). Abdominal X-ray was negative for any foreign bodies. CT of the abdomen did not show any relevant alteration. CT imaging with contrast was also unremarkable. MRI revealed restricted diffusion involving the splenium of the corpus callosum with a corresponding abnormal signal (Figure 2, Figure 3, and Figure 4). The ventricular system and basal cisterns were patent, and the visualized major intracranial vascular flow voids were intact. Moderate to marked paranasal sinus opacification with an air-fluid level within the left maxillary sinus was noted indicating potential acute sinusitis. There was no evidence of abnormal signal in the mammillary bodies or other structures near the third ventricle. All other intracranial findings were within normal limits. EEG showed no focal lateralizing or epileptiform features.

A diagnosis of MBD was made based on the MRI findings along with the clinical history of chronic alcohol and substance abuse. Although the restricted diffusion in the splenium seen on MRI may indicate an acute infarct, a lesion of chronic osmotic demyelination secondary to alcohol abuse better fits this clinical picture. 

The patient was hospitalized for five days, receiving thiamine, vitamin B complex, and folic acid. The second day after his acute change in mental status, the patient's consciousness level began to improve along with a steady regression of dysarthria and ataxia. He was discharged on the fifth day with instructions to continue taking thiamine and folic acid as well as nicotine polacrilex and a nicotine patch to aid in the detoxification process.

## Discussion

MBD is a rare neurological disorder, typically associated with chronic alcoholism, characterized by demyelination of the corpus callosum. Although the pathophysiology of MBD is not completely understood, it is hypothesized that alcohol-induced neurotoxicity plays a key role in the disease. This case contributes to the limited reports of MBD associated with polysubstance abuse, offering new insights into its pathophysiology. The clinical presentation and disease progression in this particular patient suggest that the neurotoxic effects of combined substance abuse may exacerbate the demyelination process characteristic of MBD. Alcohol neurotoxicity has been shown to alter white matter protein expression and impair lipid processing, leading to small vessel necrosis and subsequent demyelination and ischemic infarction [3]. Nutritional deficiencies, particularly of vitamin B complex, commonly found in chronic alcoholics, are also thought to contribute to the development of MBD.

The disease is more common in men than in women and usually appears between the ages of 40 and 60 [4]. MBD can be subdivided into two major subtypes based on clinical and radiological findings. Type A MBD is characterized by impairment of consciousness, hypertonia, and pyramidal signs. T2-weighted MRI shows hyperintense swelling of the corpus callosum. Type A disease has an overall poor prognosis. Type B MBD is characterized by only a slight impairment of consciousness, gait disturbances, and interhemispheric disconnection syndrome. T2-weighted MRI shows only partial hyperintensity of the corpus callosum. Type B disease has an overall favorable prognosis [5]. 

Although MBD usually involves the demyelination and necrosis of the corpus callosum, other parts of the brain have also been shown to be affected in some cases [6]. The necrotic damage disrupts the communication between the left and right hemispheres of the brain, ultimately leading to a slew of neurological symptoms. Symptoms vary widely and may include confusion, behavioral changes, dysarthria, and apraxia. Some severe cases of MBD have even resulted in coma. Neurological symptoms can fluctuate, and patients may experience a sudden onset of symptoms, such as in our patient's case. 

Diagnosis of MBD is primarily based on clinical history, neurological examination, and imaging findings. MBD is rare, and, because it lacks a typical presentation, the disease is often difficult to diagnose and differentiate from other conditions in earlier stages. The most sensitive tool for detecting characteristic changes in MBD is MRI. Recent advances in MRI development have allowed earlier diagnosis of MBD. Characteristic MRI findings include lesions in the corpus callosum, often revealing a symmetrical demyelination and cytotoxic edema [7]. The lesions usually begin in the body and later spread to the genu and the splenium, showing diffusion restriction and hyperintensity on T2 and fluid-attenuated inversion recovery (FLAIR) images. MBD-associated lesions are most often observed in the central region of the body and splenium, and the peripheral dorsal and ventral layers are usually spared (referred to as the "sandwich sign") [8]. Another characteristic finding on MRI is the "ears of the lynx" sign which consists of cone-shaped T2/FLAIR hyperintensities found at the top of the frontal horn of the lateral ventricles [9]. Chronic MBD often displays well-defined cavitary lesions [3]. Hyperintensities may also be found in various other parts of the brain, including the cerebral lobes, hemispheric white matter, and basal ganglia, although lesions in these areas often indicate more severe disease [6]. MRI is crucial in the early detection of MBD, aiding in the prompt initiation of treatment for acute cases and also in forecasting the likely outcome of the disease. 

There are a multitude of differentials that need to be addressed when making the diagnosis of MBD. Wernicke's encephalopathy (WE) and Korsakoff syndrome are neurological conditions also linked with chronic alcoholism as seen in the case of this patient. Korsakoff syndrome and WE are both caused by thiamine deficiency and affect the frontal cortex, limbic system, and thalamus [10]. MRI would most likely reveal damage to the structures around the third ventricle such as the medial nuclei of the thalamus, the tegmentum, the periaqueductal, the gray matter, or the tectal plate of the midbrain. Lesions in the mammillary bodies are most commonly seen in WE. These areas are highly dependent on oxidative metabolism and more likely to be affected by thiamine deficiency. Our patient's MRI did not show any damage in these areas making a diagnosis of WE less likely. 

There are currently no established treatment guidelines for MBD, and management mostly consists of supportive care, nutritional supplementation (particularly thiamine), and addressing any underlying alcohol or substance abuse issues. The prognosis varies; some patients may experience partial or full recovery, while others may have a progressive decline leading to severe disability or death. Early recognition and management, along with preventive measures, are crucial in managing MBD. The treatment plan for MBD parallels that of WE. Group B vitamins such as folate, thiamine, and B12 are the most commonly administered. Regardless of baseline B vitamin levels in a patient with MBD, high-dose treatment has been shown to lead to better overall outcome [1]. Hillbom et al. analyzed data from 153 cases of MBD confirmed by brain imaging and found a significant association between improved clinical outcome and thiamine treatment [11]. 

One of the limitations of this report is the lack of follow-up MR imaging. Follow-up imaging could reveal any subsequent changes in the brain lesions and help establish if complete resolution had occurred. 

## Conclusions

The diagnosis of MBD in the context of polysubstance abuse highlights the importance of considering a broad differential diagnosis in patients with complex substance use histories presenting with acute neurological changes. The influence of chronic substance use on the nervous system is not yet fully understood, and this case emphasizes the need for awareness of rare conditions like MBD as early recognition and appropriate management can significantly influence patient outcomes. This case also reveals a need for the analysis of more cases and further development of controlled studies in order to illustrate the effect of chronic substance and alcohol use on the nervous system. 
